# Metabolic and behavioral parameters of mice with reduced expression of Syndecan-1

**DOI:** 10.1371/journal.pone.0219604

**Published:** 2019-07-12

**Authors:** Christina Gougoula, Alexandra Petra Bielfeld, Sarah Jean Pour, Martin Sager, Jan-Steffen Krüssel, Wilhelm Peter M. Benten, Dunja Maria Baston-Büst

**Affiliations:** 1 Central Unit for Animal Research and Animal Welfare Affairs (ZETT) of the Heinrich-Heine-University of Düsseldorf, Düsseldorf, Germany; 2 Düsseldorf University Hospital, Department of OB/GYN and REI (UniKiD), Düsseldorf, Germany; Johns Hopkins University School of Medicine, UNITED STATES

## Abstract

Energy balance is essential for all species. Ligand-receptor interactions mediate processes that regulate body activities like reproduction and metabolism based on the energy status. Such receptors are the heparan sulfate proteoglycans and specifically the family of syndecans. Therefore we investigated the differences of metabolic parameters of heterozygous Syndecan 1 mice (*Sdc1*^*+/-*^) with reduced expression of Sdc1 and the corresponding wild type mice. *Sdc1*^*+/-*^ mice have a reduced body weight although they show increased leptin and decreased corticosterone levels. Furthermore, their food and water intake is increased. This is accompanied with less adipose tissue, smaller adipocytes and thus an increased density of adipocytes. For the detailed analysis of the metabolism the automated PhenoMaster system has been used, which allowed continuous and undisturbed recording of food and water intake, energy expenditure and movement. The reason for the lower body weight was the higher energy expenditure of these animals compared to controls. Additionally, female *Sdc1*^*+/-*^ mice showed an increased locomotor activity. Referring to organs, the intestine in *Sdc1*^*+/-*^ mice was heavier and longer, but no differences at the cellular level could be observed. These findings were independent of normal mating or *vice versa* embryo transfers of *Sdc1*^*+/-*^ and wild type embryos in recipient females of the other genotype. Herein we showed that the reduced expression of Sdc1 led to an altered metabolism on fetal as well as on maternal side, which may play a role in the growth restriction observed in human pregnancy pathologies and in mice lacking Sdc1.

## Introduction

Reproductive processes in mammals, particularly in females, require an enormous amount of energy and thus they are suppressed during times of low-energy availability. For a successful energy homeostasis and fertility maintenance an adequate communication between the hypothalamic-pituitary-gonadal axis and the peripheral metabolic status is required [1]. Metabolic sensory stimuli, hormonal mediators and neuropeptides prioritize either reproductive functions like ovulation, fertility and spermatogenesis or metabolic processes such as food intake and nutritional demands [2].

A family of cell-surface heparan sulfate proteoglycans (HSPGs), named Syndecan 1–4 (Sdc) coordinate the interactions between signaling receptors and their ligands, acting as co-receptors involved in the embryonic development [3], tumorigenesis [4], and wound healing [5]. Sdc3 which shows the closest homology to Sdc1 [6], has been studied extensively and described as a regulator of feeding behavior and body weight (BW) [7]. Furthermore, the overexpression of Sdc1 in the hypothalamic nuclei, the center of energy balance control, led to maturity-onset obesity and type-II diabetes [8]. In addition, adult homozygous *Sdc1*^*-/-*^ mice and *Sdc1*^*-/-*^ embryos were significantly lighter than their wild type (WT) littermates, regardless of their background (BALB/c or C57BL/6) [9]. In human, decreased expression of Sdc1 on the maternal site was associated with pregnancy associated pathologies like intrauterine growth restriction [10], preeclampsia [11] and hemolysis, elevated liver enzymes and low platelet count (HELLP) syndrome [12].

Within the list of chemical messengers that control food intake and reproduction are hormones that regulate the energy balance and the reproductive process and act as modulators of the intracellular availability and oxidation of glucose and free fatty acids. Initially, leptin as a product of the obese (*ob*) gene got its name from the greek word leptos which means thin, because the administration of external leptin into the *ob/ob* diabetic mouse, led to a reduced food intake and increased energy expenditure and consequently to a reduction of BW [13]. Later, adipocytes’ hormone leptin was attributed an extra ordinary role as a mediator between the body’s nutritional state and the reproductive axis. Obese and infertile ob/ob mice carry a leptin mutation in the *ob* gene and interestingly their fertility can be rescued by a leptin treatment [14, 15]. Leptin plays a critical role in the secretion of the orexigenic and anorexigenic signals. In case of a negative energy balance, low levels of leptin lead to a release of orexigenic peptides whereas high leptin levels in case of a positive energy balance, lead to increased levels of satiety neuropeptides in the hypothalamus [7]. Together with leptin, insulin acts as an adiposity signal and previous experiments have shown the role of the brain’s insulin receptor in the regulation of food intake and BW [16–18]. Apart from the well-known effects on BW, leptin is also required for a normal glucose homeostasis [19] and locomotor activity [20]. The localization of Sdc3 in the hypothalamus of WT mice and the presence of transgenically expressed Sdc1 in the hypothalamic nuclei controlling the energy balance, reveal a potential role of the Sdc proteins in the energy homeostasis [8] as co-receptors for hormones and peptide ligands [21].

Initially, it was proposed, that the adrenal hormone corticosterone plays an opposing role to insulin in the long-term regulation of the energy intake and storage [22]. Studies with rats revealed organ specific counter-rotating roles of the two hormones. In the central nervous system, insulin inhibits the food intake and corticosterone stimulates the food intake whereas in the periphery, insulin stimulates the overall energy storage whereas corticosterone inhibits the energy storage [23]. Corticosterone mediates between the energy availability and the reproductive behavior, having an increased secretion pattern in case of food deprivation and general stress combined with an inhibitory effect on reproductive processes [2]. However, studies have shown that even though glucocorticoids are secreted in response to metabolic stress, an increased concentration of these hormones is not necessarily a causal factor of metabolic challenges regarding the reproductive function [24, 25].

The aim of the present study was to investigate the metabolic situation of juvenile and adolescent *Sdc1*^*+/-*^ mice with a reduced expression of Sdc1 by analyzing blood levels of leptin, insulin, glucose and corticosterone. Furthermore, the gastrointestinal anatomy as well as the feeding and locomotive behavior of adolescent mice was examined including observations of movements and energy expenditure in the PhenoMaster cages. A complete absence of Sdc1 in human is rather rare, whereas a downregulation reflects a possible dysregulation that has been shown in human before [10, 11]. The weight of adipose tissue and intestine and intestine´s length were investigated on cellular level. In order to elicit if the maternal or the fetal *Sdc1*^*+/-*^ influence is predominant, *vice versa* experiments were performed.

## Materials and methods

### Animals

Experimental procedures as well as the maintenance of the animals were carried out in accordance to the German Legislation for the Care and Use of Laboratory animals and the EU Directive 2010/63/EU for animal experiments. Experiments were approved by the State Office for Nature, Environment and Consumer Protection (LANUV, State of North Rhine-Westphalia, Germany) (87–51.04.2010.A061, 84–02.04.2011.A317). Mice were maintained at 20–24°C on a 12 h light/12 h dark cycle with food (ssniff Spezialdiäten GmbH, Soest, Germany) and water *ad libitum* in the animal facility of the Central Unit for Animal Research and Animal Welfare Affairs (ZETT, Düsseldorf). The *Sdc1 KO* mouse was generated on a C57BL/6J background, C57BL/6J.129Sv-*Sdc1*^*tm12MB*^ [26], by completely backcrossing for 10 generations. Thereafter, we got the Sdc1 KO mouse as a kind gift from Prof. Dr. Martin Götte (University Hospital, Dept of OB/GYN, Münster, Germany). For the experiments *Sdc1*^*+/-*^ and WT mice have been used.

### Sdc1 quantification

Tail biopsies were genotyped according to the FELASA guidelines [27]. The Mouse Sdc1 ELISA Kit (biorbyt, San Francisco, California, USA) was applied for the quantitative measurement of Sdc1. Representatively, the tissue from 8 *Sdc1*^*-/-*^, 9 *Sdc1*^*+/-*^ and 25 WT mice was homogenized and lysed in tissue lysis buffer (0.5% (v/v) octylphenoxypolyethoxyethanol, 0.5% (w/v) sodium deoxycholate, 0.1% (w/v) sodium dodecyl sulfate, 50 mM Tris-HCl (pH 7.5), 150 mM NaCl, 1% (v/v) protease inhibitor cocktail (Sigma-Aldrich, Munich, Germany)). 100 μl of the homogenate was used to perform the ELISA according to the manufacturer’s instructions. Furthermore, 1 μl of the homogenate was used for whole protein quantification via BCA protein assay (Thermo Scientific, Waltham, Massachusetts, USA) to normalize the amount of Sdc1. For the analysis of independence of gender, age and degree of relationship the results of further animals were included and their number is shown in Table 1.

**Table 1:** Relative amount of Sdc1 compared to total protein for gender, age and relationship in *Sdc1*^*-/-*^, *Sdc1*^*+/-*^ and WT mice.

	Gender		Age		Degree of relationship	
	♂	♀	≤ 20 weeks	> 20 weeks	Siblings	Non-siblings
*Sdc1*^*-/-*^	0.84±0.15 (5)	1.02±0.03 (10)	1.02±0.04 (10)	0.85±0.15 (5)	0.94±0.11 (7)	0.96±0.06 (15)
*Sdc1*^*+/-*^	1.70±0.12 (6)	1.83±0.14 (11)	1.75±0.12 (13)	1.91±0.18 (4)	1.81±0.11 (6)	1.79±0.10 (17)
WT	3.87±0.30 (16)	5.10±0.77 (5)	4.36±0.37 (11)	4.69±0.99 (10)	4.91±0.38 (7)	4.52±0.50 (21)

The results are shown as mean±S.E.M. The p values were calculated for gender, age and degree of relationship for the *Sdc1*^*-/-*^, *Sdc1*^*+/-*^ as well as the WT mice and between 0.1 and 0.9 and thus not significant. The number of mice is indicated in brackets.

### *Vice versa* embryo transfer (ET)

Female mice were superovulated using 5 IU pregnant mare’s serum gonadotropin (PMSG) (Intergonan 240 IE/ml, MSD Tiergesundheit, Unterschleißheim, Germany) and 5 IU human chorionic gonadotropin (hCG) (Predalon 5000 IE, Essex Pharma GmbH, Waltrop, Germany) 48 h later, followed by mating. On day 1.5 after hCG administration, 2-cell-stage embryos were flushed out of the oviduct using M2 medium (M7167, Sigma-Aldrich, Munich, Germany). An average number of 12 2-cell embryos were transferred into the oviduct of pseudopregnant foster mothers of the opposite mouse strain (*Sdc1*^*+/-*^ embryos were transferred into 4 WT and WT embryos into 3 *Sdc1*^*+/-*^ recipients). For the ET the recipient females were anesthetized with a mixture of the anesthetic Ketaset (Zoetis, Berlin, Germany) and the sedative Rompun (Xylazin) (Bayer, Leverkusen, Germany) and analgesia was performed with Rompun and the analgesic Rimadyl (Carprofen) (Zoetis, Berlin, Germany) according to manufacturer’s instructions. Prior to ET, the recipients were mated with vasectomized males [28]. The mice born from 2 *Sdc1*^*+/-*^ and 4 WT foster mothers were weighed (Dipse digital scale TP500, Oldenburg, Germany) at day 200 and then sacrificed by cervical dislocation for organ isolation (*Sdc1*^*+/-*^ males: 8; *Sdc1*^*+/-*^ females: 6; WT males: 3; WT females: 5). The 4 fat depots and the intestinal weight (Mettler Toledo AE50, Dorsten, Germany) as well as the intestinal length of all the animals were measured.

### Blood collection and plasma analysis

Blood collection (300 μl) was performed at 6 weeks and 6 months of age with isoflurane-anesthetized mice (Table 2). We used a fine-walled capillary to slit slightly the retro-orbital sinus and subsequently collected blood in several tubes (Microvette CB 300 K2E) according to manufacturer’s instruction (Sarstedt AG & Co., Numbrecht, Germany). After centrifugation for 20 min at 2000 rpm (Universal 320R centrifuge, Hettich, Vlotho, Germany), the plasma was stored in aliquots at -20°C. Glucose was measured using a glucose oxidase assay (Glucose Assay Kit, abcam plc, Cambridge, UK). Leptin and insulin levels were quantified using a sandwich enzyme immunoassay (Leptin Quantikine ELISA, R&D Systems, Minneapolis, MN, USA and Rat/Mouse Insulin ELISA Kit, Merck Millipore, Darmstadt, Germany). Corticosterone was measured using a competitive immunoassay (Corticosterone EIA Kit, Lörrach, Germany). All assays were performed according to the manufacturer’s instructions and results are presented as mean±S.E.M.

**Table 2:** Total number of animals used for the blood analysis assays.

	Age	Gender	Glucose	Leptin	Insulin	Corticosterone
*Sdc1*^*+/-*^	6 weeks	♂	37	11	37	31
		♀	28	12	28	27
	6 months	♂	37	36	37	36
		♀	28	23	28	26
WT	6 weeks	♂	36	20	38	33
		♀	42	17	42	41
	6 months	♂	41	33	43	42
		♀	42	29	42	40

### Food and water intake

Food and water were weighed once a week for 5 weeks (Dipse digital scale TP500, Oldenburg, Germany), offered *ad libitum*, and renewed after weighing. The BW of 8 *Sdc1*^*+/-*^ and 3 WT males, as well as of 6 *Sdc1*^*+/-*^ and 5 WT females was also measured once a week and food (g/g BW) and water (ml/g BW) intake was adjusted for BW.

### The PhenoMaster system

The feeding and drinking behavior of 4 *Sdc1*^*+/-*^ and 4 WT males and 4 *Sdc1*^*+/-*^ and 4 WT females as well as their locomotion, exploration pattern and energy expenditure was tested for 3 days using the PhenoMaster system (TSE Systems, Bad Homburg, Germany), which allowed continuous and undisturbed recording [29]. Three days before the recording, the animals were placed in the room and in cages similar to the PhenoMaster cages, for their acclimatization. The following parameters were calculated: (i) daily food consumption [g] and (ii) daily water consumption [ml] as well as adjusted for BW, (iii) average distance traversed in 24 h [counts], (iv) average central and peripheral movement in 12 and 24 h [counts], (v) horizontal locomotion (x and y axis) and vertical movements (z axis) in 12 and 24 h [counts] and (vi) energy expenditure [kcal/h/g].

### Organ isolation and weighing

**Fig 1:**
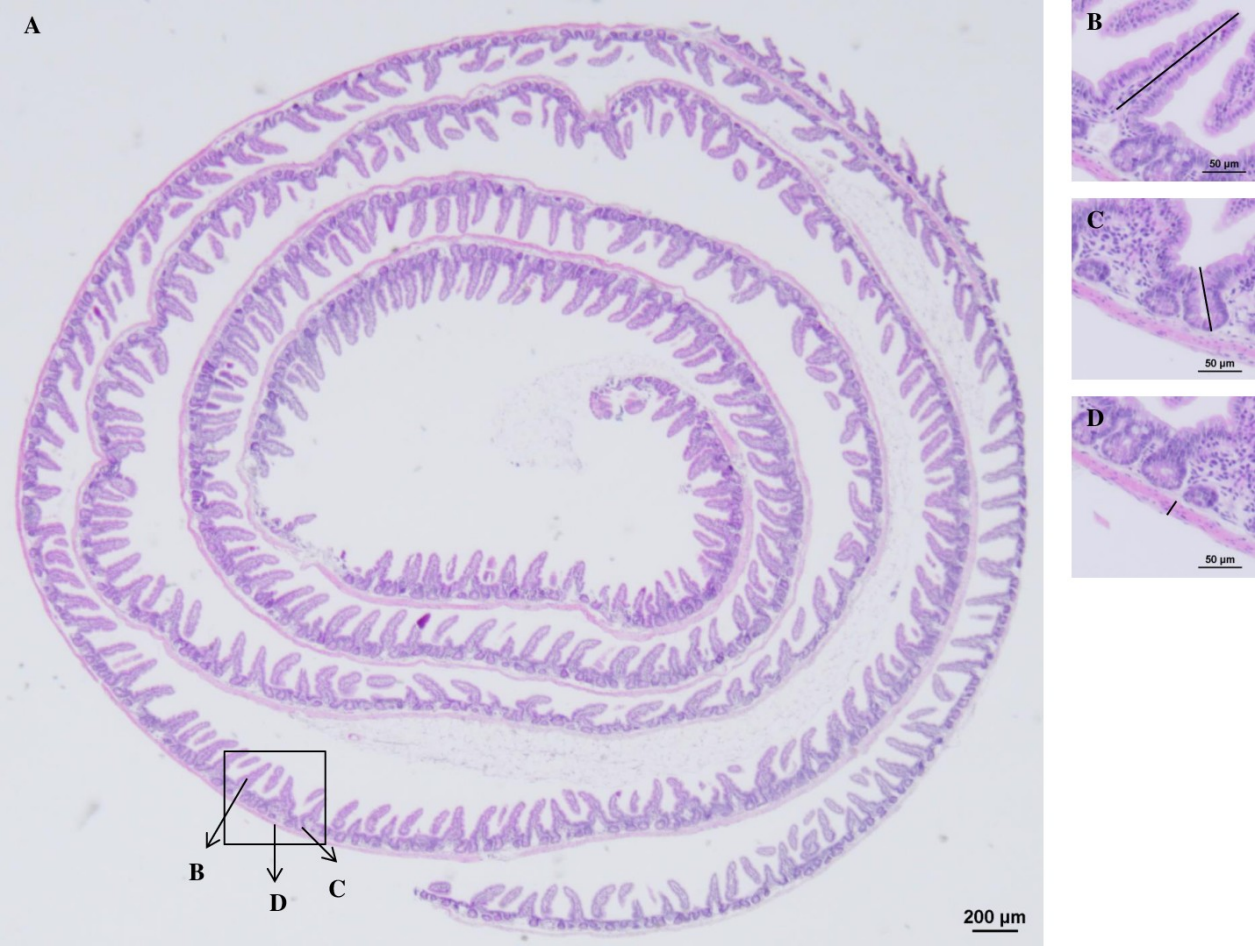
A representative Swiss-roll of the ileum of a WT female mouse. (A) The outer end of the Swiss-roll is the beginning of the intestinal part. A villus (B), a crypt (C) and a musculature site (D) are shown (length is indicated as bar).

At least 53 males and 53 females of the *Sdc1*^*+/-*^ as well as the WT group were sacrificed at the age of 6 months by cervical dislocation and weighed (Sartorius 1264 MP, Dorsten, Germany). The weight of the inguinal, gonadal, retroperitoneal and mesenteric white fat depots of 30 *Sdc1*^*+/-*^ and 30 WT animals of each gender was measured (Mettler Toledo AE50, Dorsten, Germany). The gastrointestinal tract of at least 48 *Sdc1*^*+/-*^ and at least 48 WT male as well as female mice was isolated, weighed (Mettler Toledo) and the intestinal length of 5 animals per group and per gender was measured from the pylorus to the end of the large intestine. The stomach and the cecum were then cut and emptied. The small and large intestines were gently flushed with cold PBS. The whole gastrointestinal tract was then weighed again. The small intestine was further divided into three regions: duodenum, ileum and jejunum. The jejunum was divided into 3 parts. All intestinal parts were then opened longitudinally, flattened and moisturized with 10% formalin (Sigma-Aldrich, Steinheim, Germany) before they were separately rolled with the mucosa inwards using a wooden stick according to the Swiss-roll technique introduced by Reilly & Kirsner [30]. The outer end of each Swiss-roll is the beginning of each part (Fig 1A). Processing of the intestinal tract from the sacrifice of the animals until the tissues were moisturized with formalin was completed within 5 min. The Swiss-rolls as well as the stomach, the cecum, and the fat depots were placed in an embedding cassette and immersed in 10% formalin for fixation and further histological processing. Organ to BW ratios were calculated [31–33].

### Histological and morphometrical analysis

After fixation of the tissues for 24 h with 10% formalin, samples were washed with tap water and dehydrated through a graded series of ethanol (VWR Chemicals, Leuven, Belgium) and prepared for paraffin embedding in a Tissue Tek VIP 5Jr vacuum infiltration processor (Sakura, Staufen, Germany): 50% ethanol for 15 min, 2 x in 70% ethanol for 30 min, 2 x in 96% ethanol for 60 min, 3 x in 99.5% ethanol for 60 min, 2 x in Neo-Clear (Merck, Darmstadt, Germany) for 60 min, 4 x in paraffin (Engelbrecht, Edermünde, Germany) for 45 min. Following dehydration, tissue samples were embedded in paraffin with the use of an EG 1150C modular tissue embedding system (Leica, Wetzter, Germany), cut in sections with a RM 2135 manual rotary microtome (Leica), and stained with hematoxylin (VWR Chemicals) and eosin (Merck) according to Fischer *et al*. [34]. The Swiss-rolls were cut in 4 μm and the fat pads in 5 μm thick sections and photographed with a DS-Fi3 Camera (Nikon, Düsseldorf, Germany) attached to a SMZ25 Microscope (Nikon). Each portion of the intestine was sampled according to the following protocol: starting from the outer and moving cyclically to the inner end, the length of 2 villi, the depth of 2 crypts and the thickness of 2 musculature sites were measured at each side of the Swiss-roll (up, down, right and left) using the NIS-Elements documentation Microsoft imaging software (Nikon, Version 4.60). Each Swiss-roll consisted of approx. 4–7 cycles, so that the 8 measurements per cycle resulted a total of at least 32 representative villi, crypts and musculature sites (Fig 1B, 1C and 1D). Photos of the fat pads were taken with a DS-2Mv Nikon Camera attached on an Eclipse Ti-S/Microscope (Nikon). The number and size of the adipocytes were calculated twice using the Adiposoft plug-in of the Fiji version of Image J (version 2017). For the evaluation of the fat and intestinal parameters, 5 males and 5 females of each group from normal mating as well as *vice versa* ET were processed. Only the group of the WT males that were born after the *vice versa* ET consisted of only 3 individuals.

### Statistics

Statistical analysis was performed using student’s two-tailed t-test for the Sdc1 quantification as far as the gender, the age and the degree of relationship is concerned, as well as for the weight assessment, the measurement of the metabolic parameters, the food and water consumption that was measured manually, the analysis of the PhenoMaster data, the analysis of fat weight and intestine weight and length data. In the case of the Sdc1 quantification to compare the Sdc1 amount in *Sdc1*^*-/-*^, *Sdc1*^*+/-*^ and WT animals the two-tailed t-test with Bonferroni adjustment was used. T-tests were applied using Microsoft Excel (Version: 2013). All results are depicted as mean±S.E.M. and the significances are defined as follows: *p<0.05, **p<0.02 and ***p<0.01.

## Results

### Confirmation of the reduced expression of Sdc1

**Fig 2:**
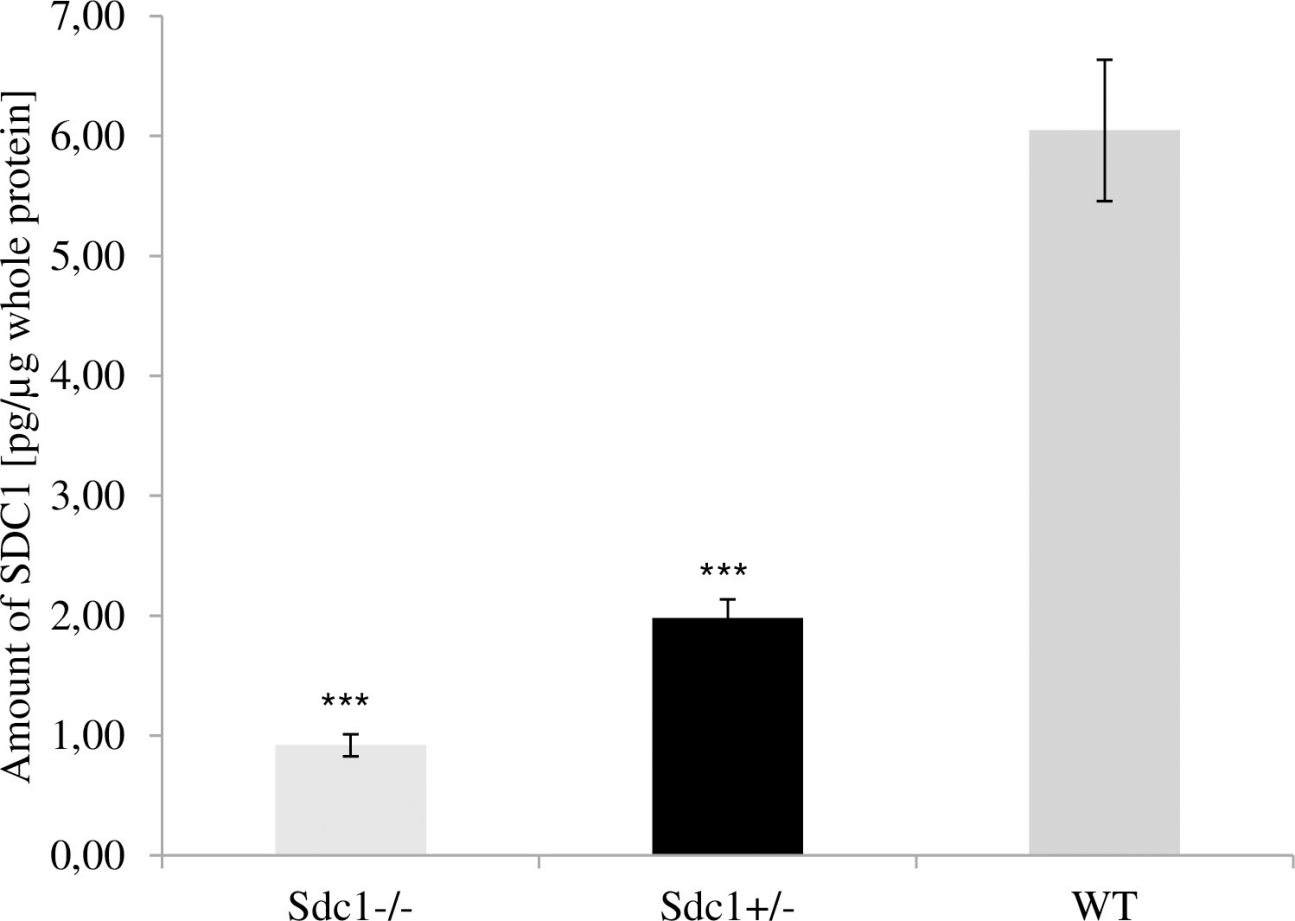
Quantification of Sdc1. Amount of Sdc1 protein in tail biopsies of *Sdc1*^*-/-*^, *Sdc1*^*+/-*^ and WT mice measured by ELISA and further normalized to the total protein (***p<0.01).

The *Sdc1*^*+/-*^ mice had a 67% significantly reduced amount of protein (Fig 2) compared to the WT animals.

The observed difference between the *Sdc1*^*+/-*^ and the WT mice was independent from gender, age and degree of relationship of the animals (Table 1).

### Weight of adult *Sdc1*^*+/-*^ and WT mice

**Fig 3:**
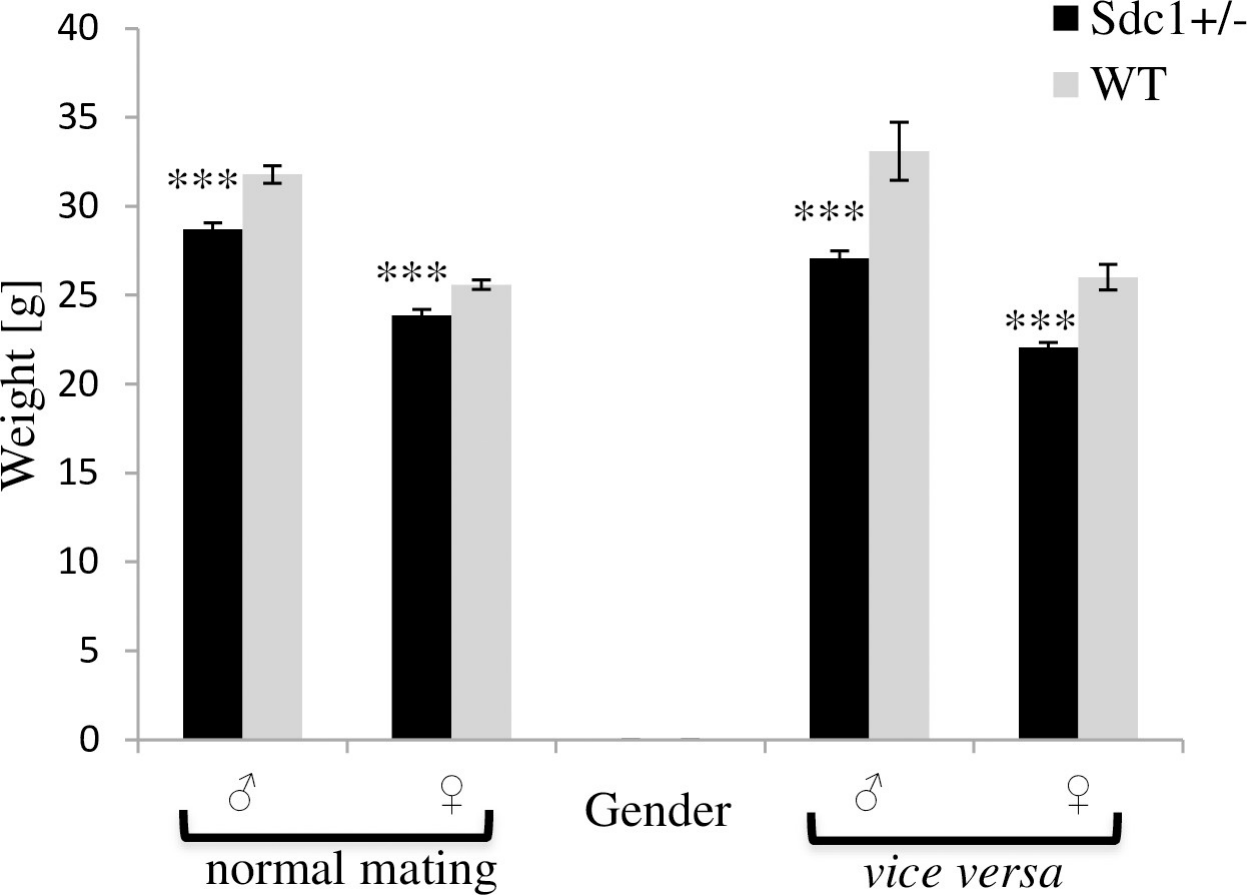
Weight assessment of adult *Sdc1*^*+/-*^ and WT males and females born either after normal mating or *vice versa* ET (***p<0.01).

At the age of 6 months, *Sdc1*^*+/-*^ and WT mice were weighed and the *Sdc1*^*+/-*^ males and females were significantly lighter (9.7%/6.8%) when compared to the WT ones (Fig 3). Additionally, animals resulting from *vice versa* ET were weighed and the *Sdc1*^*+/-*^ males and females remained significantly lighter (18.2%/15.2%) (Fig 3;) even when carried by a WT foster mother.

### Measurement of metabolic parameters

**Fig 4:**
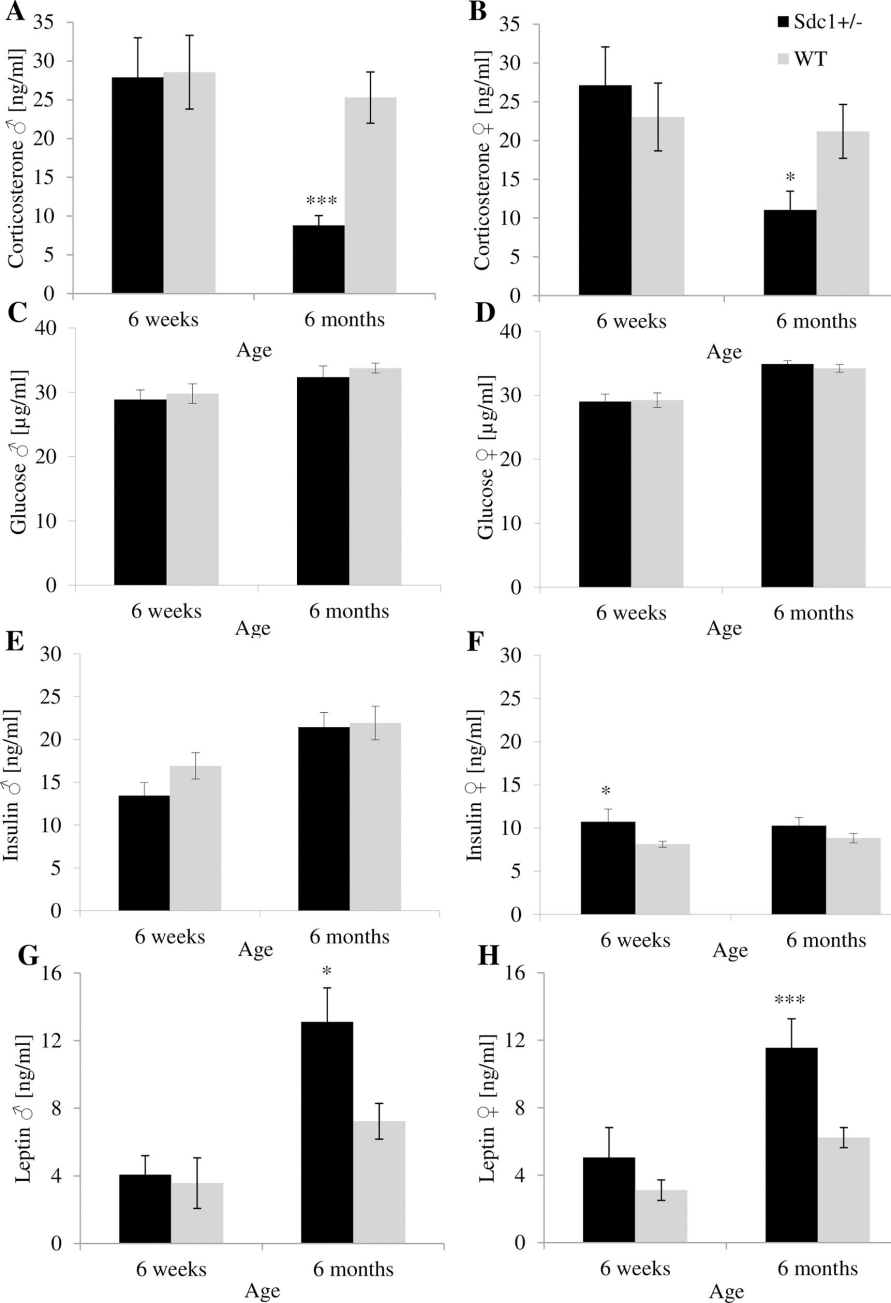
Metabolic parameters of male and female *Sdc1*^*+/-*^ and WT mice. Corticosterone (A, B), glucose (C, D), insulin (E, F) and leptin (G, H) were measured at 6 weeks and 6 months of age (*p<0.05; ***p<0.01;).

Corticosterone, glucose and leptin levels of juvenile males and females showed no significant differences between the *Sdc1*^*+/-*^ and WT mice (Fig 4). But the young *Sdc1*^*+/-*^ females had 32.1% significantly higher insulin levels compared to the WT females (Fig 4F). The adult *Sdc1*^*+/-*^ males and females showed 65.2% and 47.9% significantly lower plasma corticosterone levels compared to the WT mice (Fig 4A and 4B). Additionally, the *Sdc1*^*+/-*^ animals of both sexes showed significantly increased plasma leptin levels (male: 81.3%; female: 85.5%) in comparison to the WT animals (Fig 4G and 4H). Determination of glucose levels revealed no differences (Fig 4C and 4D).

### Manually measured food and water intake

**Fig 5:**
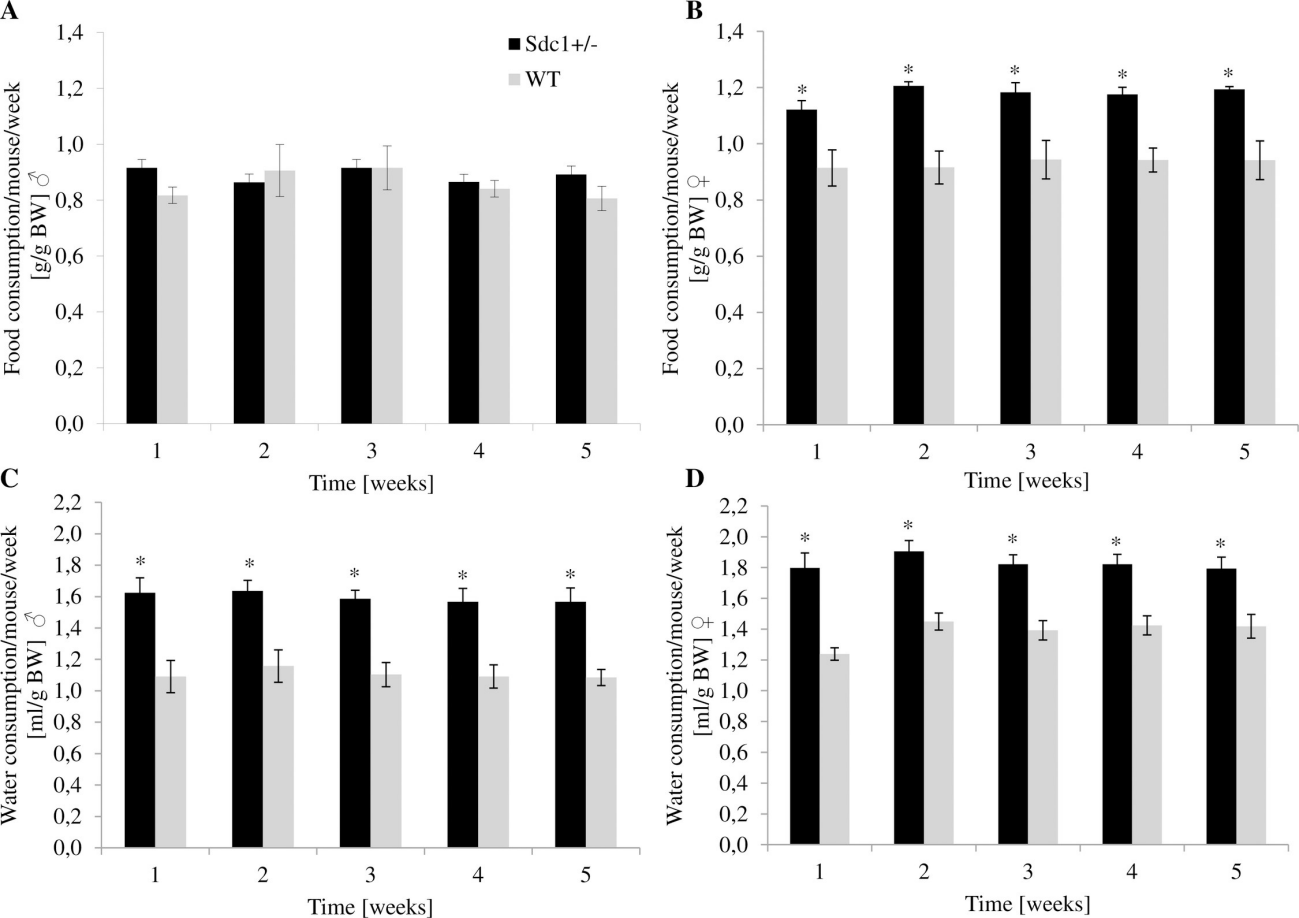
Manually measured food and water consumption of adult *Sdc1*^*+/-*^ and WT animals per week. (A, B) Average food consumption in g/g BW. (C, D) Average water intake in ml/g BW (*p<0.05).

The observed differences of corticosterone and leptin led to the measurement of food and water consumption for 5 weeks. When adjusted for BW, the *Sdc1*^*+/-*^ females ate significantly more food per week than the WT females (approx. 22%), the males only by trend (approx. 6%) (Fig 5A and 5B). Both *Sdc1*^*+/-*^ males and females drank statistically more water per week than WT animals (49% and 45% respectively) (Fig 5C and 5D).

### PhenoMaster cage activity

**Fig 6:**
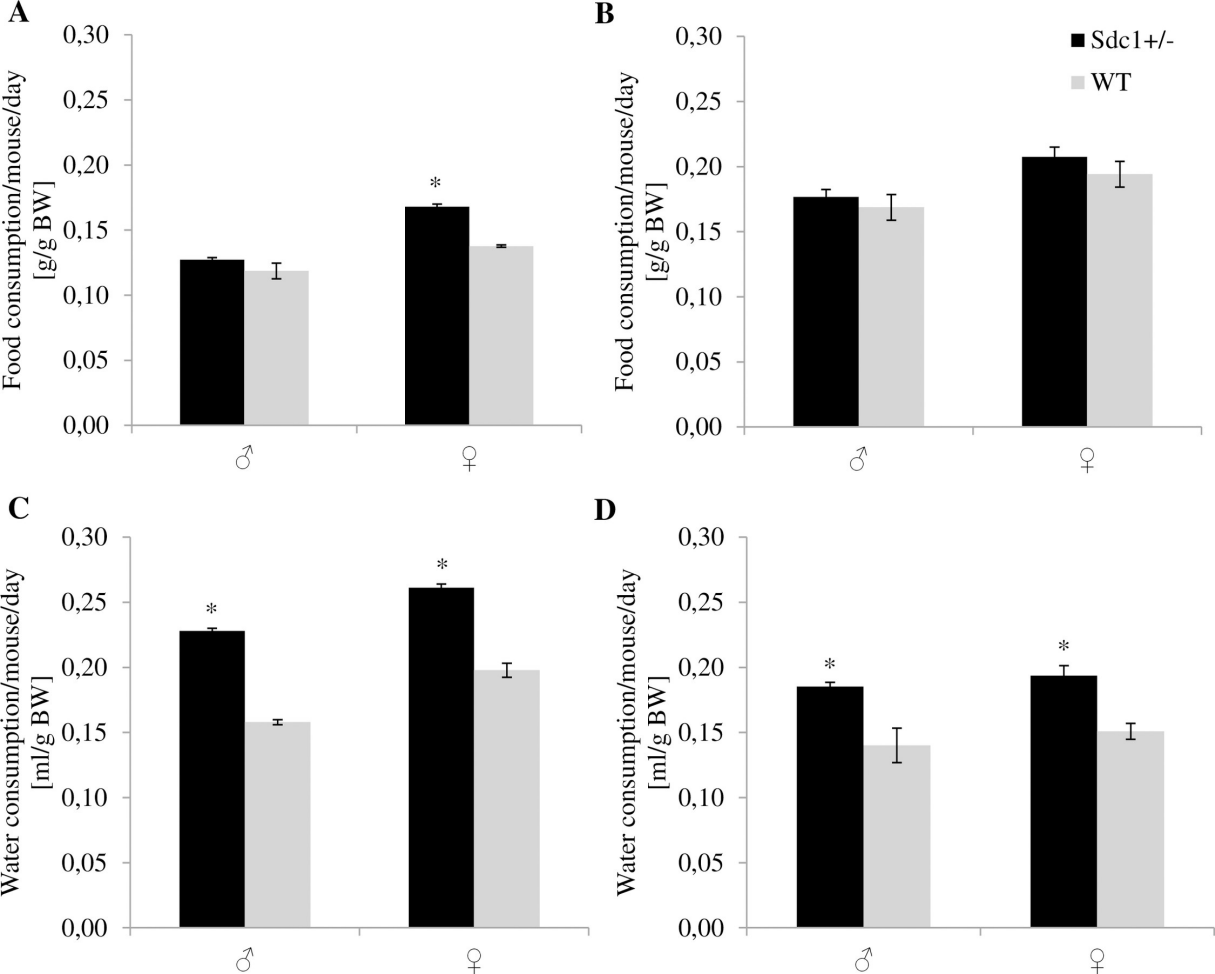
Comparison of manually and PhenoMaster automatically measured food and water consumption of adult *Sdc1*^*+/-*^ and WT animals per day. (A) Manually measured average food consumption in g/g BW. (B) Average food consumption in g/g BW determined using the PhenoMaster system. (C) Manually measured average water consumption in ml/g BW. (D) Average water consumption in ml/g BW determined using the PhenoMaster system (*p<0.05).

Data obtained for the nutritional measurements were in congruence with the food and water consumption of the animals mentioned above (Fig 6).

The overall activity pattern of the *Sdc1*^*+/-*^ and WT mice was recorded continuously by the PhenoMaster cages for 3 consecutive days, however due to acclimatization only the data of the last 56 h have been analyzed. No significant differences could be observed for the distance and the speed of the animals (males: *Sdc1*^*+/-*^: 378.32 m; WT: 482.35 m and females: *Sdc1*^*+/-*^: 637.95 m; WT: 505.32 m; *Sdc1*^*+/-*^ males 20.63±4.15 cm/s within 24 h, WT males 26.94±4.56 cm/s, *Sdc1*^*+/-*^ females 38.69±5.42 cm/s and WT 30.38±6.00 cm/s).

**Fig 7:**
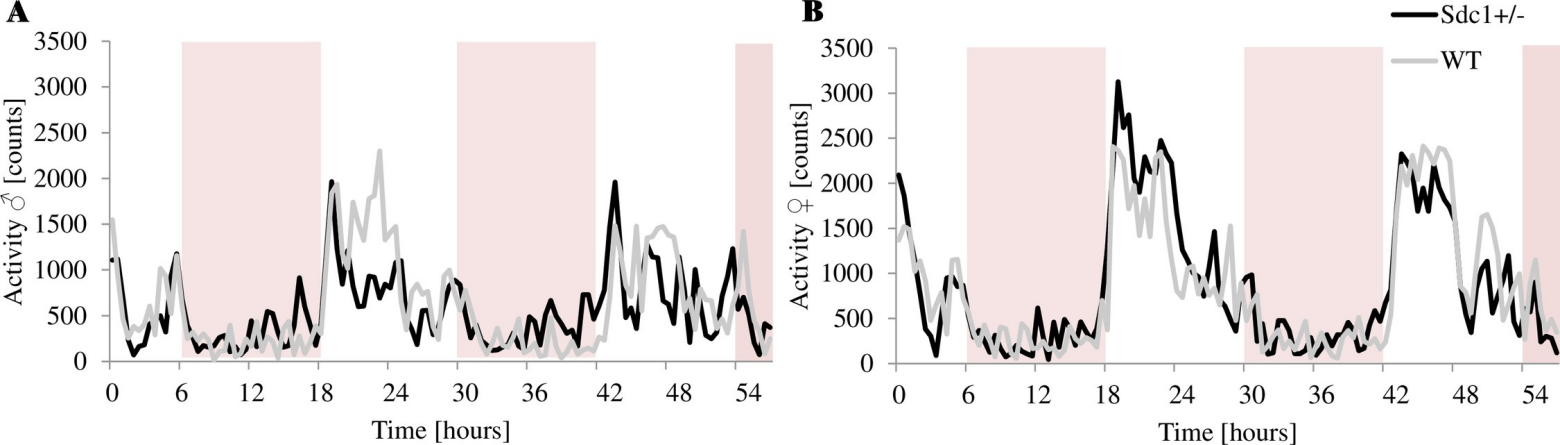
The activity profile of *Sdc1*^*+/-*^ and WT male and female mice measured by the PhenoMaster system within 56 h. (A, B) Circadian activity of the *Sdc1*^*+/-*^ and the WT male and female mice (shaded area indicates light hours 6 a.m.– 6 p.m.). The activity pattern shown was identical for the x, y and/or z axis as well as the activity at the center and at the periphery of the cage.

**Fig 8:**
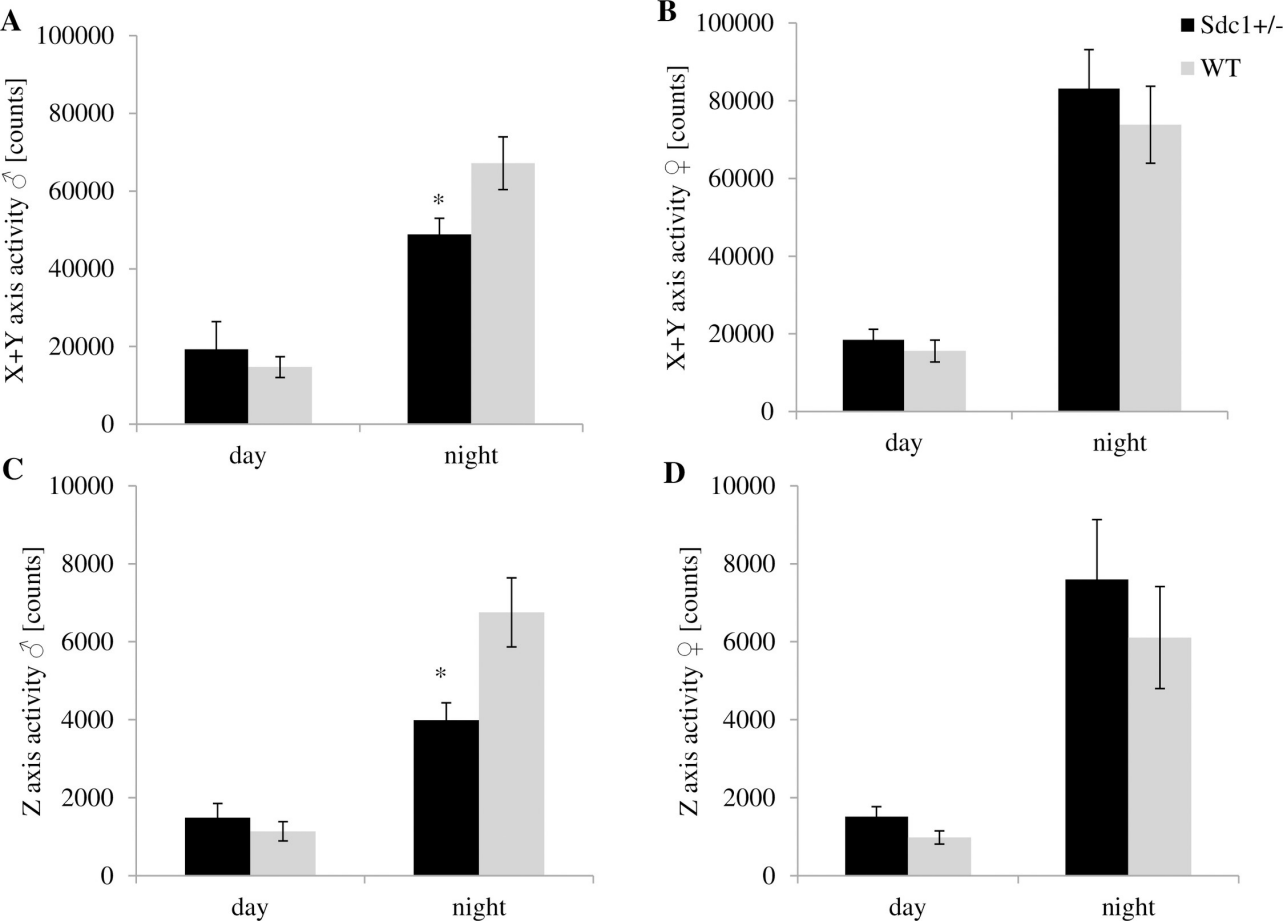
The activity of *Sdc1*^*+/-*^ and WT male and female mice measured by the PhenoMaster system. The average activity of two days is shown divided into day and night. (A, B) The average activity on the x and y axis. (C, D) The average activity on the z axis (*p<0.05).

**Fig 9:**
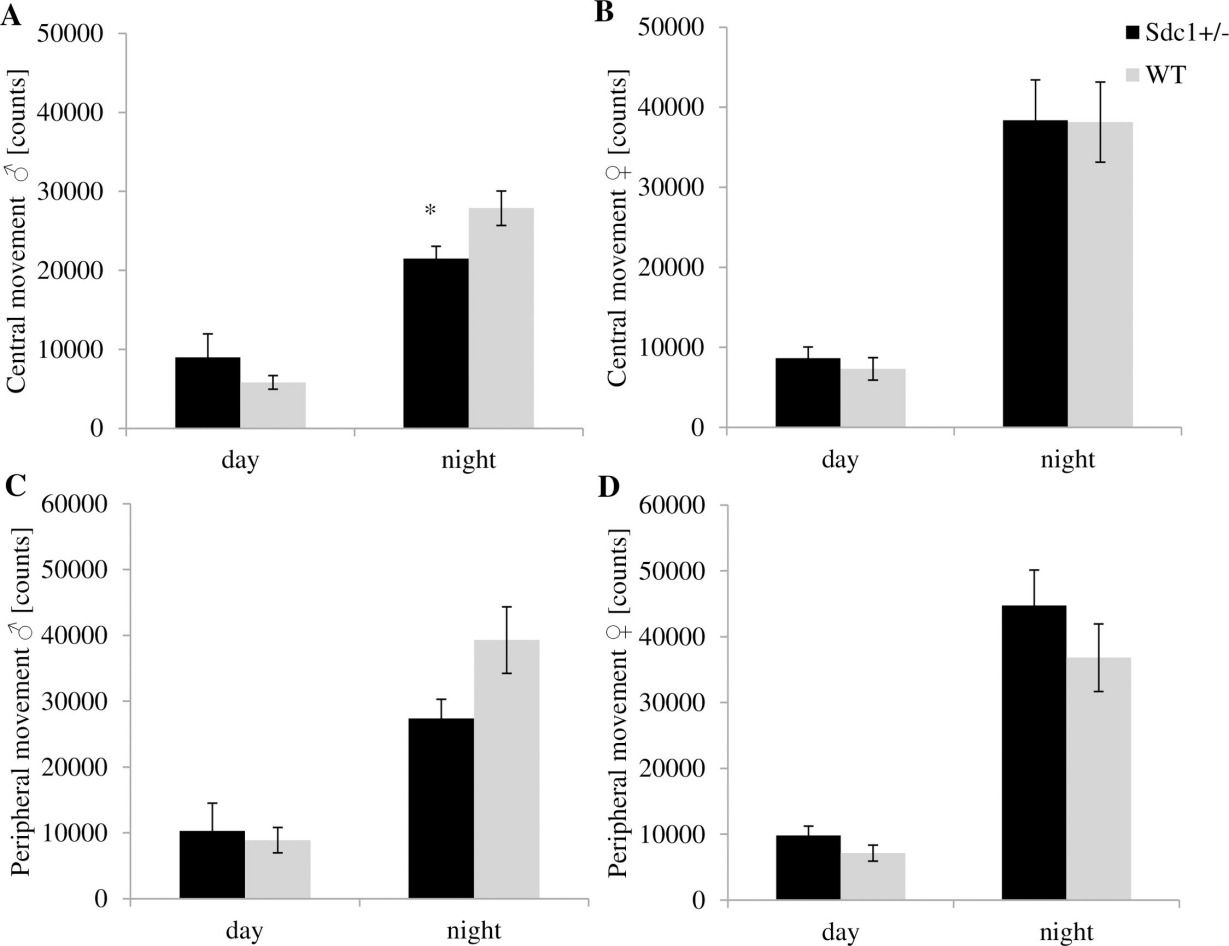
Central and peripheral activity of *Sdc1*^*+/-*^ and WT male and female mice measured by the PhenoMaster system. The average activity of two days is shown divided into day and night. (A, B) The average activity in the center of the cages. (C, D) The average activity in the periphery of the cages (*p<0.05).

**Fig 10:**
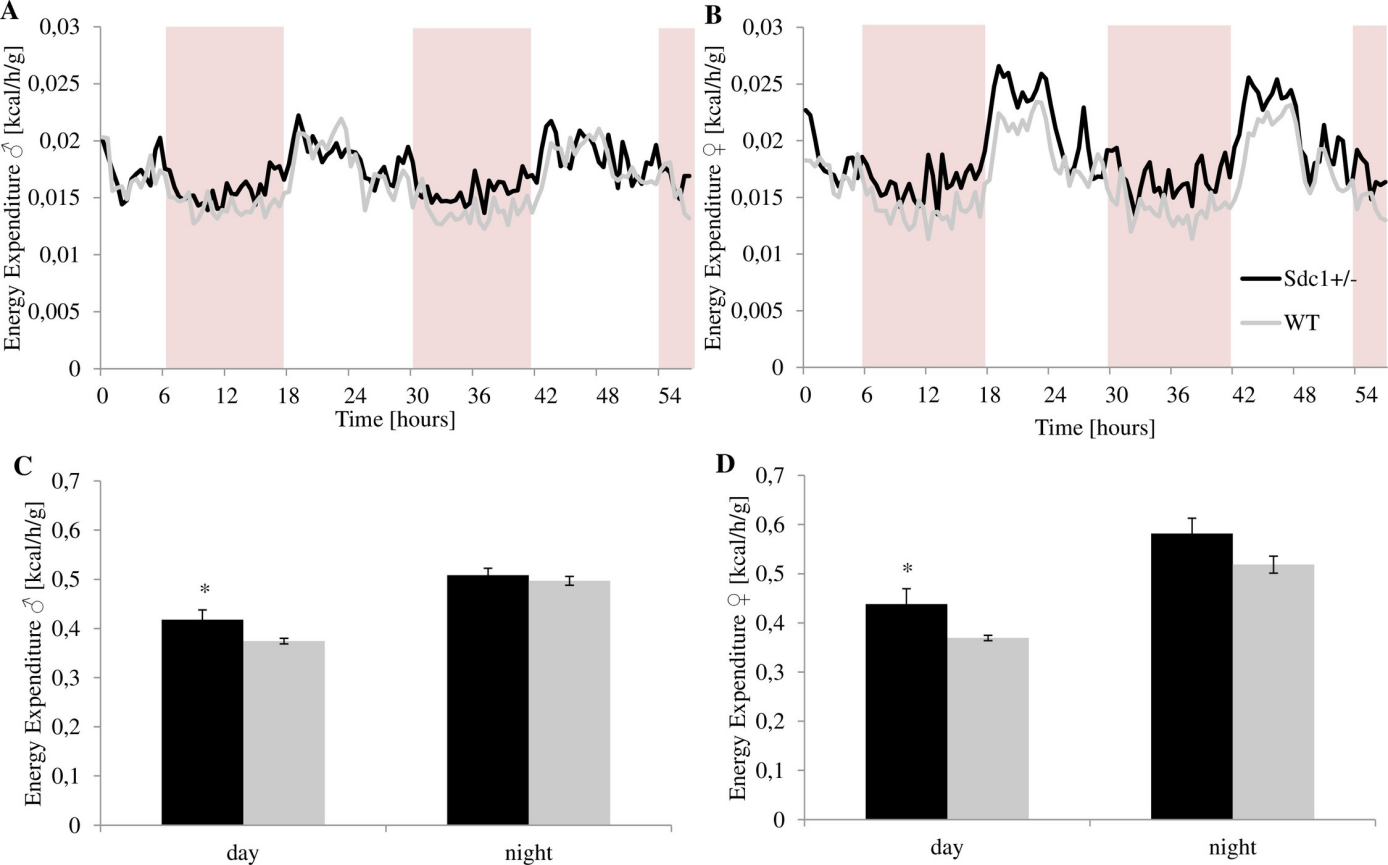
Energy expenditure of the *Sdc1*^*+/-*^ and WT males and females measured by the PhenoMaster system. (A, B) The energy expenditure profile was measured within 56 h (shaded area indicates light hours 6 a.m.– 6 p.m.). (C, D) The average energy expenditure of two days is shown divided into day and night (*p<0.05).

As expected for nocturnal animals, the kinetic was considerably higher during the dark phase than during the light phase with a circadian time course. The locomotion was analyzed with regard to central and/or peripheral movements as well as movements on the x, y and/or z axis In general, it could be stated that the *Sdc1*^*+/-*^ females were more and the males less active than the WT mice. During the night, the *Sdc1*^*+/-*^ males were significantly calmer than the WT males. The *Sdc1*^*+/-*^ males are more active at the end of the day phase and at the beginning of the night period. On the contrary, the WT males show their main activity in the middle of the night phase (Fig 7). The *Sdc1*^*+/-*^ animals of both sexes were by trend more active during the day, during the night the *Sdc1*^*+/-*^ males were significantly less active than the WT males and the *Sdc1*^*+/-*^ females by trend more active than the WT ones (Fig 8). As far as the movement at the center as well as at the periphery of the cage is concerned, the Sdc1^+/-^ animals of both sexes were by trend more active during the day, whereas by the night, the WT males moved significantly more in the center and by trend in the periphery than the Sdc1^+/-^ males and the *Sdc1*^*+/-*^ females moved slightly more in the periphery than the WT females (Fig 9). Completing the movement profile, the energy expenditure during the day was significantly higher for both male and female *Sdc1*^*+/-*^ mice (11.6% and 18.6%) compared to WT (Fig 10).

### Calculation of the fat depots

**Fig 11:**
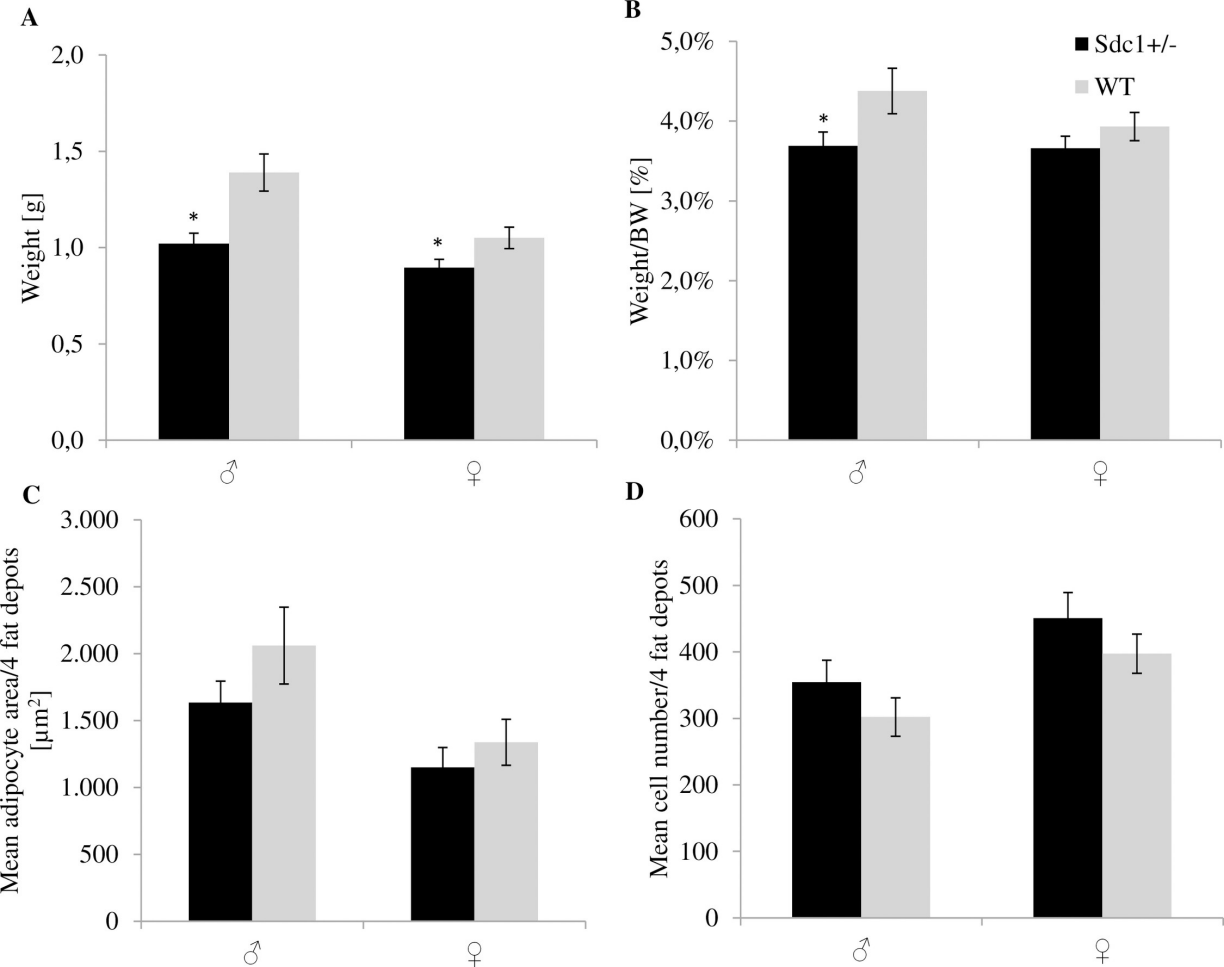
Parameters of the examination of the fat depots of the *Sdc1^+/-^* and WT animals that were born after normal mating. (A) Fat weight of inguinal, retroperitoneal, mesenteric and gonadal depots of *Sdc1*^*+/-*^ and WT male and female mice in [g] and (B) adjusted for BW. (C, D) The mean adipocyte area [**μ**m^**2**^] and the total number of adipocytes per microscopic field of view of the 4 fat depots of *Sdc1*^*+/-*^ and WT males and females (*p<0.05).

**Fig 12:**
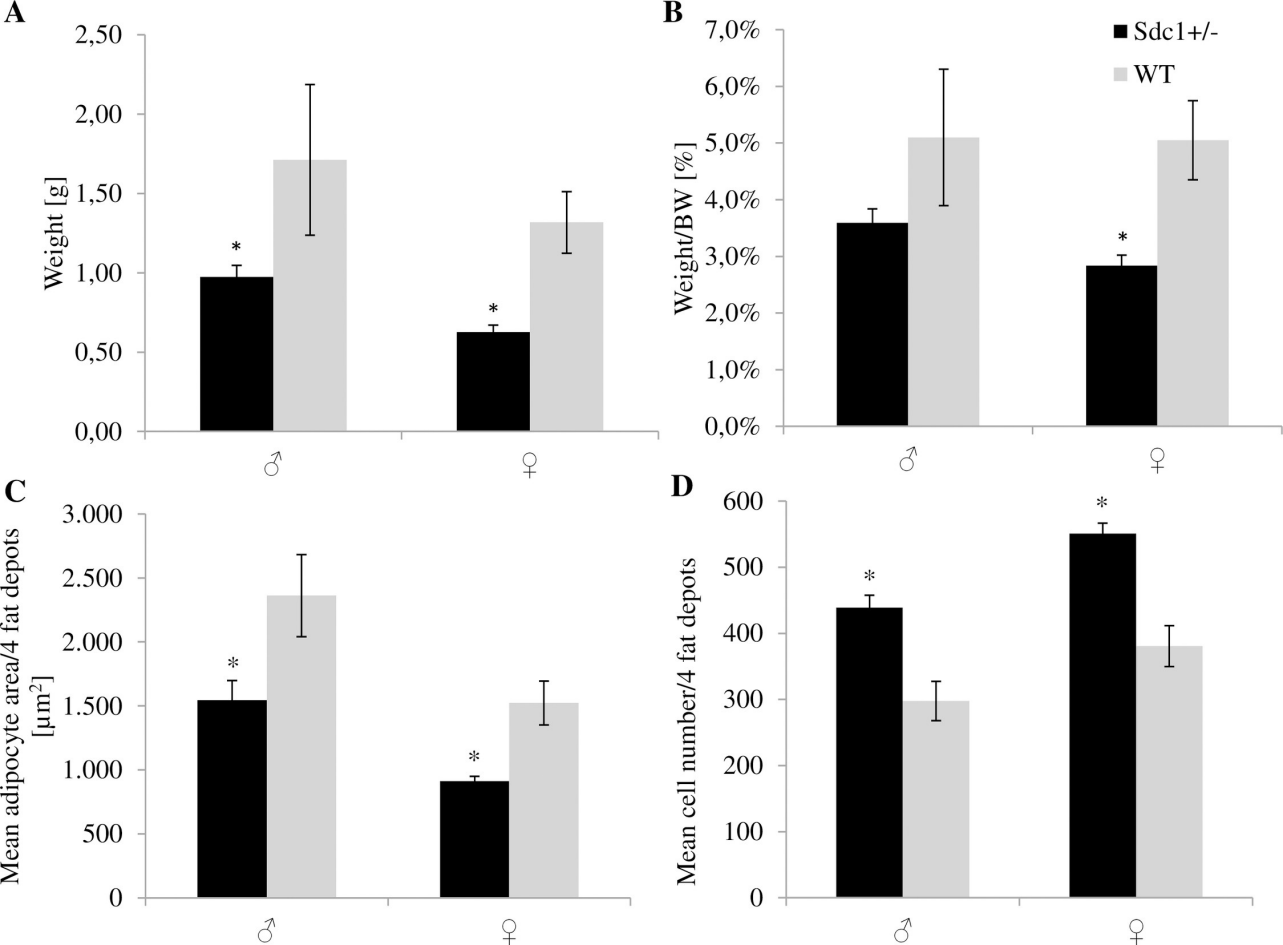
Parameters of the examination of the fat depots of the *Sdc1^+/-^* and WT animals that were born after vice versa embryo transfer. (A) Fat weight of inguinal, retroperitoneal, mesenteric and gonadal depots of *Sdc1*^*+/-*^ and WT male and female mice in [g] and (B) adjusted for BW. (C, D) The mean adipocyte area [**μ**m^**2**^] and the total number of adipocytes per microscopic field of view of the 4 fat depots of *Sdc1*^*+/-*^ and WT males and females (*p<0.05).

**Fig 13:**
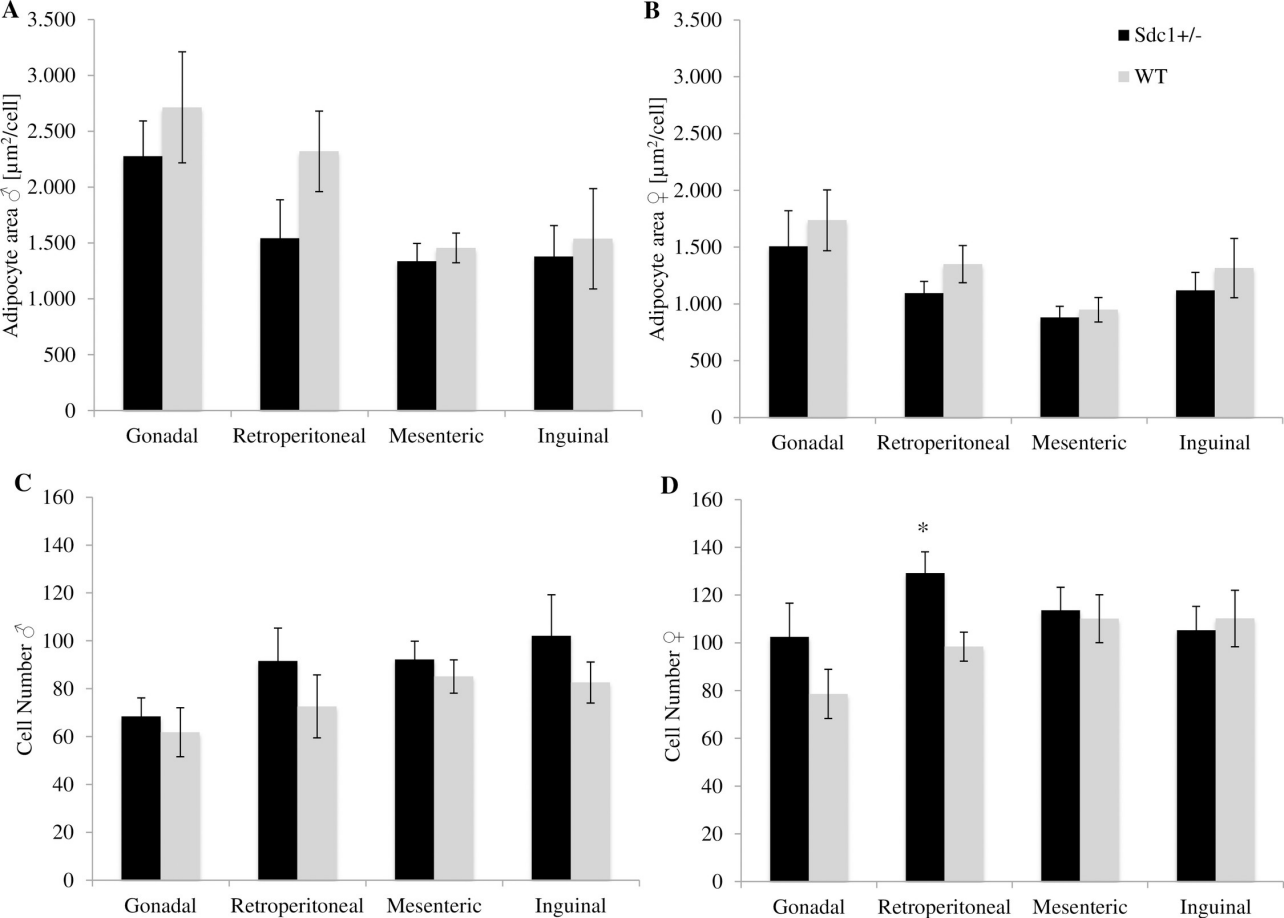
Parameters of the examination of each fat depot of the *Sdc1^+/-^* and WT animals that were born after normal mating. (A, C) The mean adipocyte area and number per microscopic field of view isolated from *Sdc1*^*+/-*^ and WT male and (B, D) female mice (*p<0.05).

**Fig 14:**
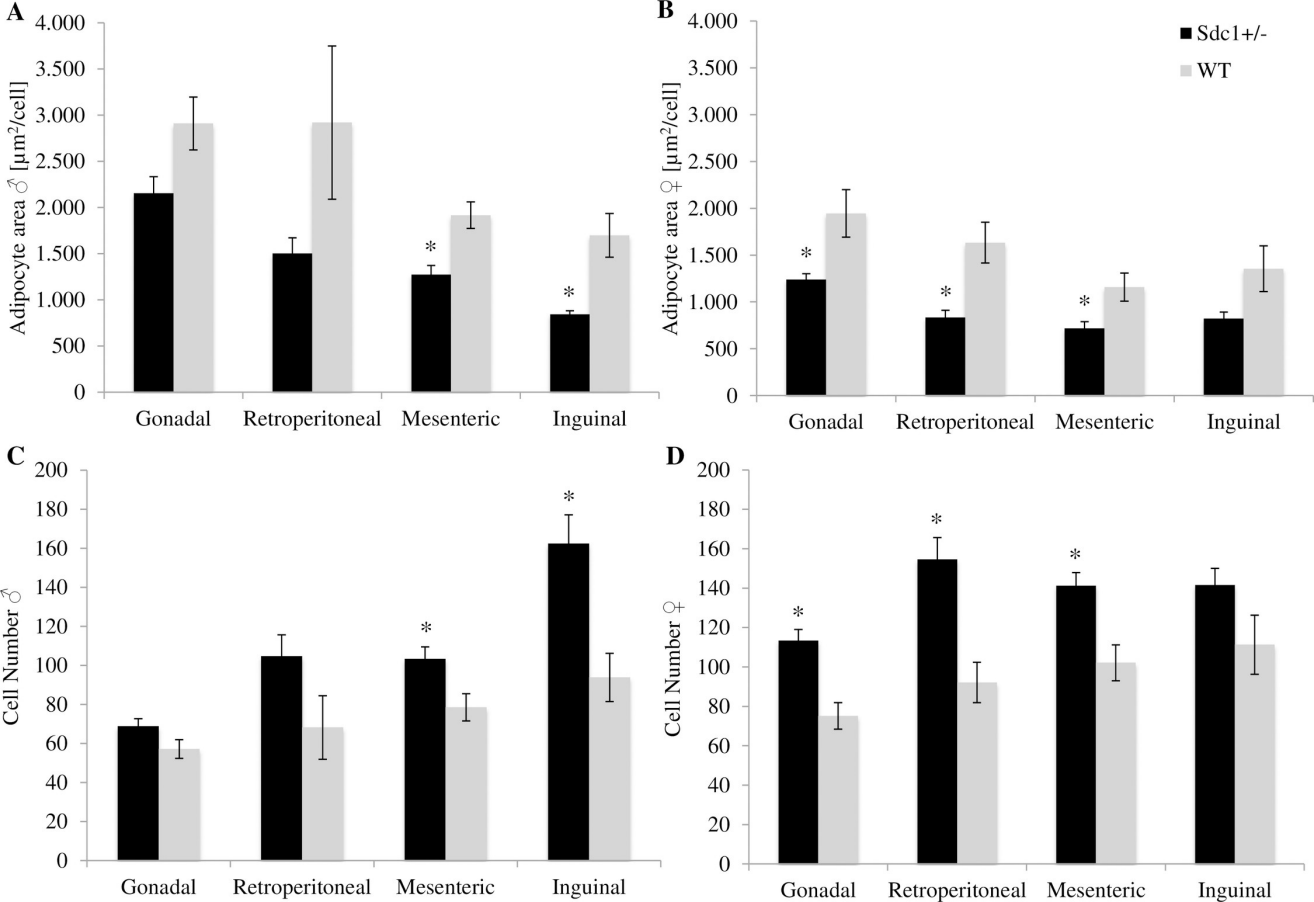
Parameters of the examination of each fat depot of the *Sdc1^+/-^* and WT animals that were born after vice versa embryo transfers. (A, C) The mean adipocyte area and number per microscopic field of view isolated from Sdc1^**+/-**^ and WT male and (B, D) female mice (*p<0.05).

Isolation and excision of the 4 white fat depots (inguinal, gonadal, retroperitoneal and mesenteric) revealed that the *Sdc1*^*+/-*^ male and female adult mice had significantly less fat (26.6% and 14.8%) than the WT animals referring to the absolute weight (Fig 11A) as well as the relative values (Fig 11B). Moreover, the *Sdc1*^*+/-*^ males and females resulting from *vice versa* ET had 43.1% and 52.5% significantly less fat compared to WT animals (Fig 12A and 12B). The above mentioned differences led to the examination at cellular level. The adipocytes of each fat depot and each gender presented smaller but more adipocytes partly by trend per microscopic field of view (Figs 11C, 11D [normal mating], 12C and 12D [*vice versa*]) independent from their origin (Figs 13 and 14).

### Observation of intestinal weight and length

**Fig 15:**
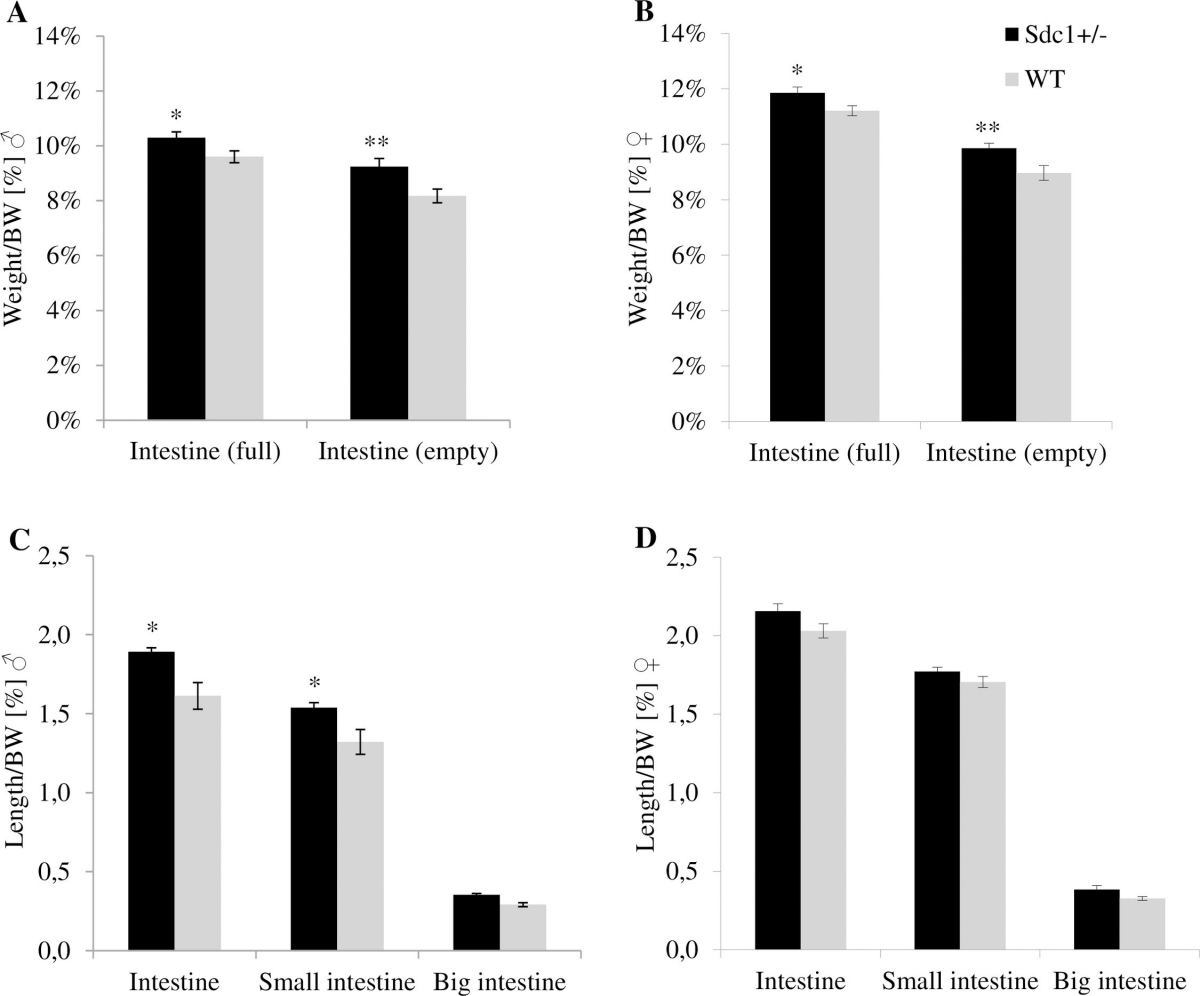
Relative intestinal weight and length differences of adult *Sdc1*^*+/-*^ and WT males and females after normal mating. (A, B) The intestinal weight before (full) and after (empty) removal of the intestinal content. (C, D) The intestinal length is shown for the whole intestine and for the intestinal parts of the small and the big intestine (*p<0.05, **p<0.02).

**Fig 16:**
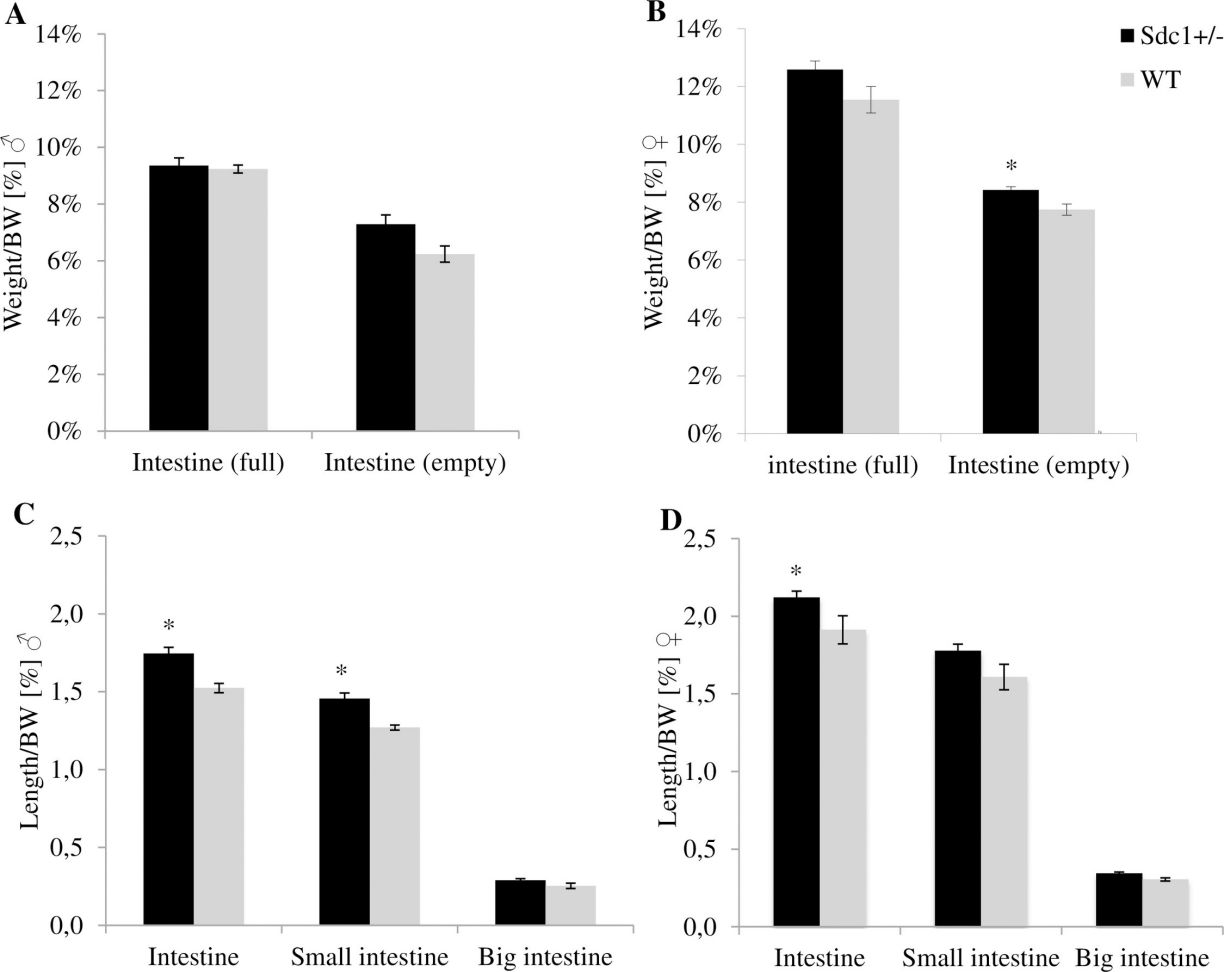
Relative intestinal weight and length differences of adult *Sdc1*^*+/-*^ and WT males and females after *vice versa* embryo transfers. (A, B) The intestinal weight before (full) and after (empty) removal of the intestinal content. (C, D) The intestinal length is shown for the whole intestine and for the intestinal parts of the small and the big intestine (*p<0.05).

The intestinal weight of adult *Sdc1*^*+/-*^ and WT males and females resulting from normal mating and *vice versa* ET was measured before and after the intestinal content was removed. Independent from gender and origin, *Sdc1*^*+/-*^ mice had a significantly higher relative weight for the full intestine (normal mating male 7.3%, female 5.8%, *vice versa* male 1.3%, female 9.1%) as well as for the empty intestine (normal mating male 13.1%, female 10.0%, *vice versa* male 16.9%, female 8.7%) than the WT mice, as shown in Fig 15A and 15B for normal mating and in Fig 16A and 16B for *vice versa* embryo transfer. Intestinal differences represented in weight could also be depicted in length. The *Sdc1*^*+/-*^ males either born by *Sdc1*^*+/-*^ mothers (Fig 15C) or by WT foster mothers (Fig 16C) had significantly longer intestines than the corresponding WT males (normal mating 17.3%, *vice versa* 14.6%). Only by trend differences could be observed for the females born by *Sdc1*^*+/-*^ mothers (Fig 15D), whereas the *Sdc1*^*+/-*^ females resulting from *vice versa* ET showed significantly longer intestines than the corresponding WT females (Fig 16D) (normal mating 6.2%, *vice versa* 10.9%).

The intestine were examined microscopically for the entire length of each intestinal part (duodenum, jejunum, ileum, colon, rectum) using the Swiss-roll technique (Fig 1). No significant differences for villi, crypts and intestinal musculature were found between *Sdc1*^*+/-*^ and WT animals within the five different intestinal sections independent from gender and origin.

## Discussion

Maintenance of the energy balance determines the dynamics between physiological processes such as reproduction and metabolism and can be an indicator of the organism’s health status. Concentrations of circulating metabolic factors and neuroendocrine reproductive indicators are modulated by secreted and cell-surface molecules such as the HSPGs. More specifically, the Sdc family has been linked to reduced metabolic rates and body size in *Drosophila melanogaster* before [35]. It was shown that the overexpression of Sdc1 in the mouse hypothalamus led to hyperphagic and obese mice [8]. In our study, the reduction of Sdc1 led to significantly lighter mice compared to WT animals, as it has been shown also by a previous study of our team [36]. This weight reduction is in accordance with previous findings in *Sdc1*^*-/-*^ mice either with the same (C57BL/6J) or another (BALB/c) background [9]. In addition, mammary ductal development was shown to be impaired in these mice as well [37]. However, the altered lactation as a reason for this postnatal growth restriction has already been studied with already prenatal smaller *Sdc1*^*-/-*^ embryos [9]. In addition, in our study, the smaller habitus remained even after *vice versa* ET.

Focusing on the hormonal effects, the *Sdc1*^*+/-*^ male and female mice showed increased leptin levels and less plasma corticosterone. Elevated leptin levels have also been reported in transgenic mice overexpressing Sdc1, whereas corticosterone levels remained unaffected [8]. By common physiological theories, leptin and corticosterone act inversely to regulate the energy balance: leptin decreases whereas corticosterone increases food intake [38, 39]. The *Sdc1*^*+/-*^ mice in our study behaved completely contradictory towards normal physiology. The higher energy expenditure due to an increased movement of *Sdc1*^*+/-*^ mice could shed a light at this metabolic phenotype. An increased food consumption and a higher energy expenditure combined with a reduced BW and no increased storage in the fat depots, has been described in the literature for a fibroblast growth factor transgenic mouse model before [40]. The *Sdc1*^*-/-*^ mice have been reported to be cold stressed in normal housing conditions because of a disruption of the intradermal adipose tissue development [41]. It is possible that the *Sdc1*^*+/-*^ mice consume more food and show an increased energy expenditure, in order to counterbalance the energy demands and to overcome the cold stress. Therefore it would be beneficial to examine the energy expenditure of *Sdc1*^*+/-*^ mice in slightly warmer housing conditions and when fed with a high fat diet. Furthermore, male and female mice with a reduced expression of Sdc3 showed a resistance to the weight gain caused by a high-fat diet and also had a significantly increased energy expenditure during day and night [42]. Higher leptin levels could be associated with a boost of small adipocytes in leptin overexpressing mice being in congruence with our data for the *Sdc1*^*+/-*^ mice [43]. It was assumed that this population of small adipocytes might expand rapidly and lead to an ob phenotype. Focusing on Sdc1, which is expressed during adipocyte differentiation [41, 44], it was shown before that the leptin expression is highly correlated with the adipocyte size and number [45] which might explain higher leptin levels in case of small adipocytes. Furthermore, a positive correlation between the leptin gene expression and adipocyte volume has been suggested only in the well-nutritioned state, whereas in the case of fasting or negative energy balance, this relationship might be interrupted [45]. In order to answer the question if the higher need for calories and the lower BW impairs the intestine of *Sdc1*^*+/-*^ males and females, its habitus and cellular composition were examined. During tissue preparation and intestinal evacuation a more watery stomach content was observed in the case of the *Sdc1*^*+/-*^ animals, which is in agreement to their higher water consumption. A positive correlation between food and water intake has already been proved [46, 47]. The absence of Sdc1 led to an increased intestinal protein leakage in both human and mice, but Bode *et al*. suggested different mechanisms for barrier function and junction formation or maintenance and the influence of Sdc1 [48, 49]. For the maintenance of a balance between the energy intake and the energy utilization, the adipose tissue is activated, where the energy from diets is stored and from there further used for basic cellular functions and physical activities [50]. The white adipose tissue senses the energy state of the body and stores energy as triglyceride in lipid droplets in case of energy excess or breaks down triglyceride to free fatty acids into the circulation when energy is needed [51]. On the other hand the principal role of the brown adipose tissue is to dissipate energy in the form of heat, a process called thermogenesis, through a mechanism called uncoupled respiration mediated by uncoupling protein-1 [52]. However, white adipose tissue possesses the ability to acquire characteristics of brown fat in response to thermogenic stimuli, which has been associated with *in vivo* cold tolerance and increased energy expenditure [53], which is of great importance if the cold stress situation of the *Sdc1*^*-/-*^ mice will be taken under consideration.

Previous studies of our group have demonstrated a correlation between reduced levels of Sdc1 and the reproductive phenotype [36]. *Sdc1*^*+/-*^ mice present an impaired reproductive phenotype and a genotype-related growth restriction [36], which is in accordance to the literature, where pregnancy associated pathologies have been described in relation to a reduced expression of Sdc1 [10–12]. The present study focuses on the altered metabolism as a result of a reduced expression of Sdc1, which can contribute to the growth restriction observed in pregnancy pathologies in human and mice [9–11]. Further experimentation will shed light on the metabolic pathways and cellular and hormonal events of the current findings and will answer the question whether a clinical routinely detection of low levels of Sdc1 is possible and an according supplementation would cure those pathologies.

## Supporting information

S1 File Full data availability. (XLS)

## Abbreviations

BW: body weight
ET: embryo transfer
hCG: human chorionic gonadotropin
HSPG: heparan sulfate proteoglycan
KO: knock out
Ob: obese
PBS: phosphate-buffered salt solution
PMSG: pregnant mare’s serum gonadotropin
Sdc: Syndecan
WT: wild type
